# Inclisiran: A New Promising Agent in the Management of Hypercholesterolemia

**DOI:** 10.3390/diseases6030063

**Published:** 2018-07-13

**Authors:** Constantine E. Kosmas, Alba Muñoz Estrella, Andreas Sourlas, Delia Silverio, Elizabeth Hilario, Peter D. Montan, Eliscer Guzman

**Affiliations:** 1 Department of Medicine, Division of Cardiology, Mount Sinai Hospital, New York, NY 11357, USA; 2 Department of Medicine, Mount Sinai St. Luke’s-West Hospital, New York, NY 10025, USA; alba.munoz.estrella@gmail.com; 3 School of Medicine, University of Crete, 71003 Heraklion, Greece; med3553@edu.med.uoc.gr; 4 Cardiology Clinic, Cardiology Unlimited, PC, New York, NY 10033, USA; deliasg10@gmail.com (D.S.); bethhilario@hotmail.com (E.H.); drpetermontan@gmail.com (P.D.M.); 5 Department of Medicine, Division of Cardiology, Montefiore Medical Center, Bronx, NY 10467, USA; eliscer@hotmail.com

**Keywords:** proprotein convertase subtilisin-kexin type 9 (PCSK9), small interfering RNA (siRNA), inclisiran, low-density lipoprotein-cholesterol (LDL-C), cardiovascular disease (CVD), cardiovascular risk

## Abstract

The discovery of proprotein convertase subtilisin-kexin type 9 (PCSK9), a serine protease which binds to the low-density lipoprotein (LDL) receptors and targets the receptors for lysosomal degradation, offered an additional route through which plasma LDL-cholesterol (LDL-C) levels can be controlled. Initially, the therapeutic approaches to reduce circulating levels of PCSK9 were focused on the use of monoclonal antibodies. To that effect, evolocumab and alirocumab, two human monoclonal antibodies directed against PCSK9, given on a background of statin therapy, have been shown to markedly decrease LDL-C levels and significantly reduce cardiovascular risk. The small interfering RNA (siRNA) molecules have been used recently to target the hepatic production of PCSK9. siRNA interferes with the expression of specific genes with complementary nucleotide sequences by affecting the degradation of mRNA post-transcription, thus preventing translation. Inclisiran is a long-acting, synthetic siRNA directed against PCSK9 and it has been shown to significantly decrease hepatic production of PCSK9 and cause a marked reduction in LDL-C levels. This review aims to present and discuss the current clinical and scientific evidence pertaining to inclisiran, which is a new promising agent in the management of hypercholesterolemia.

## 1. Introduction

Cardiovascular disease (CVD) is the number one cause of death worldwide. An estimated 17.7 million people died from CVD in 2015, representing 31% of all global deaths [1]. Hypercholesterolemia is a major known risk factor for cardiovascular disease and low-density lipoprotein cholesterol (LDL-C) lowering has been unequivocally shown to cause a significant reduction in cardiovascular risk in both primary and secondary prevention [2,3]. Statins are the standard of care and have a proven efficacy in LDL-C lowering and in the reduction of CVD risk [4,5]. However, there is considerable variability in individual responses to statins [6] and many individuals at risk for CVD fail to achieve LDL-C goals [7,8]. Furthermore, several patients demonstrate intolerance to statins, mostly due to myalgias and weakness [9]. These factors necessitate research for the development of new additional therapies with a favorable side effect profile that would improve our ability to achieve LDL-C goals and decrease CVD risk [10].

The discovery of proprotein convertase subtilisin-kexin type 9 (PCSK9) in 2003 [11], a serine protease which binds to the LDL receptors and targets the receptors for lysosomal degradation, thereby reducing their recycling and decreasing the removal rate of circulating LDL-C [12], offered an additional route through which plasma LDL-C levels can be controlled [13]. Initially, the therapeutic approaches to reduce circulating levels of PCSK9 were focused on the use of monoclonal antibodies. To that effect, evolocumab and alirocumab, two human monoclonal antibodies directed against PCSK9, given on a background of statin therapy, have been shown to markedly decrease LDL-C levels and significantly reduce cardiovascular risk [14,15]. Circulating PCSK9 is generated mainly by the liver; hence, therapeutic agents curtailing the hepatic production of PCSK9 may provide an alternative to the use of monoclonal antibodies. Inclisiran is a chemically synthesized small interfering RNA (siRNA) molecule, which targets the hepatic production of PCSK9 and is currently under investigation for its LDL-C lowering effect and potential for cardiovascular risk reduction.

## 2. siRNAs: Mechanism of Action

siRNAs are ~20–30 nucleotide RNA molecules, which lately have emerged as critical regulators in the expression and function of eukaryotic genomes. These molecules, which may be active in both the somatic and germline lineages of different eukaryotic species, are involved in the regulation of endogenous genes and in the protection of the genome from invasive nucleic acids [16]. In most mammalian cells, long double-stranded RNA induces an interferon response, which contributes to the antiviral defense. This interferon response prompts a generalized shutdown of protein synthesis. As a result, long double-stranded RNA cannot be used for specific gene silencing. On the contrary, siRNAs can evade the radar of the mammalian interferon response and produce strong and specific gene silencing [17]. More specifically, siRNA interferes with the expression of specific genes with complementary nucleotide sequences by affecting the degradation of mRNA post-transcription, thus preventing translation [18].

## 3. Inclisiran

The siRNA molecule has been used recently to decrease PCSK9 levels. The siRNA molecules follow the natural pathway of RNA interference (RNAi) by binding intracellularly to the RNA-induced silencing complex (RISC), thus enabling it to cleave messenger RNA (mRNA) molecules specifically encoding PCSK9 [19].

Inclisiran is a long-acting, synthetic siRNA directed against PCSK9, which is conjugated to triantennary *N*-acetylgalactosamine carbohydrates. These carbohydrates bind to abundant liver-expressed asialoglycoprotein receptors, leading to the uptake of inclisiran specifically into the hepatocytes [20,21].

**Figure 1:**
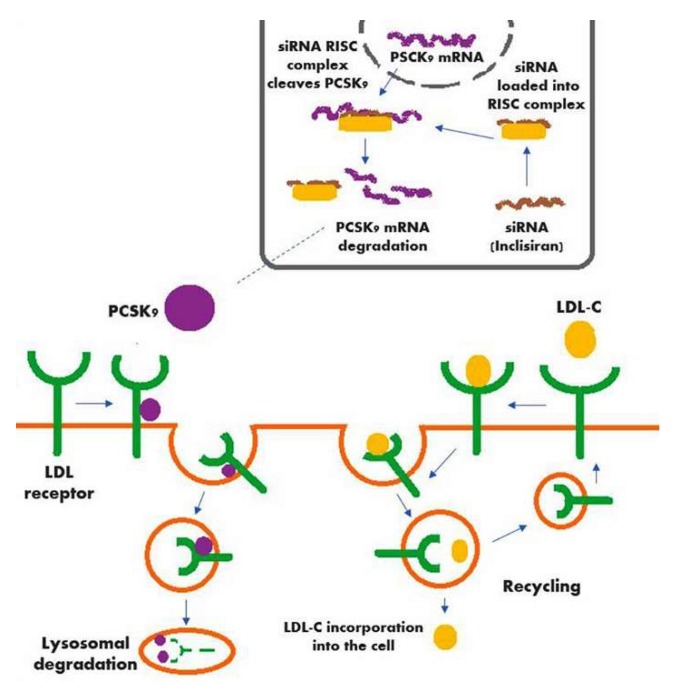
Mechanism of action of inclisiran in conjunction with the action of PCSK9.

A schematic of the mechanism of action of inclisiran (along with the action of PCSK9, the hepatic production of which is significantly decreased by inclisiran) is shown in Figure 1.

## 4. Clinical Trials with Inclisiran

In a randomized, single-blind, placebo-controlled, phase 1 dose-escalation study in healthy adult volunteers with serum LDL-C levels ≥ 3.00 mmol/L (116 mg/dL), participants were randomly assigned in a 3:1 ratio to receive one dose of intravenous ALN-PCS (inclisiran), with doses ranging from 0.015 to 0.400 mg/kg, versus placebo. In the group of participants who received treatment with inclisiran at the dose of 0.400 mg/kg, there was a significant mean reduction in circulating PCSK9 and LDL-C levels by 70% and 40%, respectively, as compared with placebo (*p* < 0.0001 for both comparisons). The proportions of patients affected by treatment-emergent adverse events were similar in the inclisiran and placebo groups (79% vs. 88%, respectively) [22].

In another phase 1 trial, several different doses of inclisiran or placebo were administered subcutaneously to healthy volunteers with an LDL-C level of at least 100 mg/dL. The doses of inclisiran tried were single-dose-injections (25, 100, 300, 500, and 800 mg) or multiple-dose injections (125 mg weekly for four doses, 250 mg every other week for two doses, and 300 or 500 mg monthly for two doses) with or without concurrent statin therapy. Single doses of inclisiran ≥ 300 mg decreased the PCSK9 level by up to a least-squares mean reduction of 74.5% from baseline to day 84, whereas single doses of ≥100 mg lowered LDL cholesterol-C by up to a least-squares mean reduction of 50.6% from baseline. Reductions in the PCSK9 and LDL-C levels were maintained at day 180 for doses of ≥300 mg. All multiple-dose regimens of inclisiran reduced the levels of PCSK9 and LDL-C by up to a least-squares mean reduction of 83.8% and 59.7% from baseline to day 84, respectively. There were no serious adverse events observed with inclisiran and the most common adverse events were cough, musculoskeletal pain, nasopharyngitis, headache, back pain, and diarrhea [23].

ORION-1 was a phase 2, multicenter, double-blind, placebo-controlled, multiple-ascending-dose trial of inclisiran, administered as a subcutaneous injection, in patients at high risk for CVD with LDL-C levels > 70 mg/dL in the presence of a history of atherosclerotic CVD (ASCVD) or > 100 mg/dL in the absence of a history of ASCVD. A total of 501 patients were randomly assigned to receive a single dose of placebo or 200, 300, or 500 mg of inclisiran, or two doses of placebo or 100, 200, or 300 mg of inclisiran at days 1 and 90. The primary end point was the change in LDL-C level from baseline level at 180 days. At enrollment, 73% of the patients were on statin therapy, and 31% of the patients were on treatment with ezetimibe. At day 180, the least-squares mean reductions in LDL-C levels were 27.9 to 41.9% in the patients who received a single dose of inclisiran and 35.5% to 52.6% in the patients who received two doses (*p* < 0.001 for all comparisons vs. placebo). The greatest reduction in LDL-C levels was attained with the two-dose 300-mg inclisiran regimen and 48% of the patients who received that regimen had an LDL-C level < 50 mg/dL at day 180. Furthermore, the two-dose 300-mg inclisiran regimen caused a least-squares mean reduction in PCSK9 levels by 69.1% (*p* < 0.001 vs. placebo) and decreased high sensitivity C-reactive protein (hsCRP) by 16.7% (*p* < 0.05). Serious adverse events occurred in 11% of the patients who received inclisiran and in 8% of the patients who received placebo. Injection-site reactions occurred in 4% of patients who received one dose and in 7% of patients who received two doses of inclisiran [24]. Thus, in this phase 2 trial, inclisiran produced significant reductions in LDL-C and PCSK9 levels with an acceptable side effect profile, as compared to placebo. However, given the relatively small number of patients and the short duration of the study, no definitive conclusions can be drawn regarding the long-term side effect profile of inclisiran and further larger studies with a longer follow-up period are required for that reason.

ORION-11 is an ongoing placebo-controlled, double-blind, randomized, phase 3 trial with inclisiran. The study will enroll individuals with ASCVD or ASCVD-risk equivalents and elevated LDL-C despite maximum tolerated dose of LDL-C lowering therapies in order to evaluate the efficacy, safety, and tolerability of subcutaneous inclisiran injection(s). The study will be an international multicenter study (non-United States). Currently, 1617 participants have been enrolled. The patients will be randomly assigned to either inclisiran or placebo. Doses of 300 mg of inclisiran sodium (equivalent to 284 mg of inclisiran) will be administered as subcutaneous injections on day 1, day 90, and then every 6 months. Primary outcomes include percentage change in LDL-C from baseline to day 510 and time-adjusted percent change in LDL-C levels from baseline between day 90 and day 540. The secondary outcomes include absolute change in LDL-C from baseline to day 510, time-adjusted absolute change in LDL-C from baseline between day 90 and day 540, as well as the percentage changes in the levels of PCSK9, total cholesterol, apolipoprotein B (ApoB), and non-high-density lipoprotein cholesterol (non-HDL-C) from baseline to day 510 [25].

ORION-11 trial is one of the four phase 3 pivotal trials with inclisiran. Others include the ORION-10 trial with approximately 1500 ASCVD patients in North America, the ORION-9 trial with approximately 400 patients with heterozygous familial hypercholesterolemia (FH) in North America, Europe, Israel, and South Africa, as well as the ORION-5 trial with 60 patients with homozygous FH in Europe, Middle East, and North America [26].

## 5. Conclusions and Future Directions

Medical interventions targeting PCSK9 have emerged as a very promising therapeutic strategy in the management of hyperlipidemia. By inhibiting the expression of PCSK9, significant reductions in the levels of LDL-C have been obtained that may lead to a reduction of cardiovascular risk [20]. As it was mentioned before, two PCSK9 inhibitors (evolocumab and alirocumab) have already been tested in large outcome trials and have been shown to significantly decrease cardiovascular risk [14,15].

On the other hand, inclisiran, a synthetic siRNA molecule, has been shown to significantly decrease hepatic production of PCSK9 and cause a marked reduction in LDL-C levels [22,23,24], although, up to date, there are no available studies showing the effect of inclisiran on intermediate CVD markers, such as intima-media thickness of the carotid artery (CIMT), arterial flow-mediated dilation (FMD), or arterial pulse wave velocity (PWV). Advantages of inclisiran over monoclonal antibodies directed against PCSK9 include its infrequent administration (twice a year vs. 12–26 injections per year for PCSK9 inhibitors), as well as the fact that anti-PCSK9 monoclonal antibodies act at a plasma level, whereas inclisiran acts at the intracellular level of hepatocytes to mitigate the levels of LDL-C and PCSK9 [27].

Furthermore, inclisiran appears to have a relatively benign side effect profile, as shown in the ORION-1 trial. There were only rare symptoms of immune activation, such as flu-like symptoms, which is often a concern with RNA-targeting therapies. In addition, inclisiran did not adversely affect platelet levels, in contrast to other recent reports from studies of antisense oligonucleotides and other siRNA molecules [24,28]. However, the ongoing ORION-11 trial [25] is expected to better define the long-term side effect profile of inclisiran.

Notwithstanding, it is not yet ascertained that the marked LDL-C reductions attained with inclisiran would definitely translate into a reduction in CVD risk, and thus, large outcome trials would need to be conducted in the future for that reason.

Other potential future therapeutic strategies targeting PCSK9, which are currently in the initial stages of development, include small molecule inhibitors that disrupt the processing of PCSK9, the use of adnectins, which block the binding of PCSK9 to the LDL receptor [20,29], as well as the AT04A vaccine, which is currently being tested in a phase 1 clinical trial [20,30].
